# Human Thelaziasis, Europe

**DOI:** 10.3201/eid1404.071205

**Published:** 2008-04

**Authors:** Domenico Otranto, Moreno Dutto

**Affiliations:** * University of Bari, Bari, Italy; † Croce and Carle Hospital, Cuneo, Italy

**Keywords:** Thelazia callipaeda, eyeworm, human thelaziasis, dogs, cats, zoonosis, Phortica variegata, vector, distribution, Europe, dispatch

## Abstract

*Thelazia callipaeda* eyeworm is a nematode transmitted by drosophilid flies to carnivores in Europe. It has also been reported in the Far East in humans. We report *T*. *callipaeda* infection in 4 human patients in Italy and France.

Nematodes transmitted by arthropods may cause diseases of different severity, especially in developing countries (*1*). Among these nematodes, *Thelazia callipaeda* Railliet and Henry, 1910 (Spirurida, Thelaziidae) has received little attention. Commonly referred to as eyeworm, it infects orbital cavities and associated tissues of humans, carnivores (i.e., dogs, cats, and foxes), and rabbits (*2*). Because of its distribution in the former Soviet Union and in countries in the Far East, including the People’s Republic of China, South Korea, Japan, Indonesia, Thailand, Taiwan, and India (*2*) it has been known as oriental eyeworm. *T*. *callipaeda* infection is endemic in poor communities in Asia, particularly in China (*3*), where it is frequently reported as being responsible for human thelaziasis with mild to severe symptoms (including lacrimation, epiphora, conjunctivitis, keratitis, and corneal ulcers) (*4*).

A second species, *T*. *californiensis* Price, 1930, has been reported to infect humans in the United States (*2*). Infective third-stage larvae of eyeworm are transmitted by insects that feed on lacrimal secretions of infected animals and humans that contain *Thelazia* spp. first-stage larvae. In the vector *T*. *callipaeda*, first-stage larvae undergo 3 molts (≈14–21 days), and infective third-stage larvae may be transmitted to a new receptive host and develop into the adult stage in ocular cavities within ≈35 days (*5*). Competence of drosophilid flies of the genus *Phortica* as vectors of *T*. *callipaeda* has been recently demonstrated (*6*–*8*).

Ocular infection of carnivores by *T*. *callipaeda* has been reported in France (*9*). This infection is also common in dogs (Figure 1) and cats in Italy (*10*). Imported carnivore cases of thelaziasis have also been reported in Germany, the Netherlands, and Switzerland (*11*). The number of case reports of human thelaziasis has increased in several areas of Asia (*3*), where it occurs predominantly in rural communities with poor living and socioeconomic standards and mainly affects the elderly and children. In spite of increasing reports of *T*. *callipaeda* infection in carnivores in different European countries, no human cases have been described. Thus, infection with this eyeworm is unknown to most physicians and ophthalmologists.

**Figure 1:**
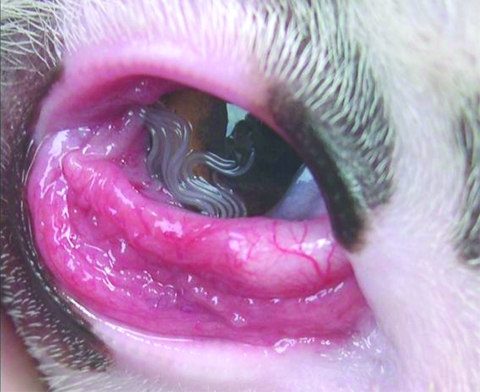
Massive *Thelazia callipaeda* eye infection in a dog.

We report autochthonous cases of human thelaziasis in Europe. We sought to raise awareness in the scientific community of the risk for disease caused by this parasite and the need to include this infection in the differential diagnosis of ocular diseases.

## The Study

From June 2005 through August 2006, a total of 4 patients with human thelaziasis were referred to the Department of Emergency and Admissions at Croce and Carle Hospital in Cuneo, Italy, for consultation. The 4 male patients (age range 37–65 years) lived in northwestern Italy (43°N, 6°E) and southeastern France (46°N, 9°E), where infections had been reported in dogs, cats, and foxes (*9*,*12*). All patients had similar symptoms (exudative conjunctivitis, lacrimation, and foreign body sensation) for a few days to weeks before referral (Table). All patients required medical attention during the summer (June–August 2005 and 2006) and reported floating filaments on the eye surface. A medical history was obtained for 3 of the patients. The other patient (patient 2, a homeless man) was referred to a physician at the local social services in Nice, France, for severe mental disorders, poor hygiene, and diabetes (Table). Infections in patient 2 were diagnosed 1 month apart in each eye (June and July 2005; referred to as patient 2a and 2b). None of the patients had had any eye disease or had traveled outside their area of residence, with the exception of patient 1 who had gone trekking in the woods in Tenda (Piedmont region, Italy) ≈3 weeks before the onset of symptoms.

**Table:** Male patients with human thelaziasis, Europe, 2005–2006

Patient no.	Date of symptoms	Location	Age, y	Infected eye	No. and sex of nematodes
1	2005 Jun	Roja Valley, Liguria, Italy	45	Right	1 fourth-stage larva
2a	2005 Jun	Nice, France	65	Right	2 females, 1 male
2b	2005 Jul	Nice, France	65	Left	1 male
3	2006 Aug	Canelli, Piedmont, Italy	48	Right	1 male
4	2006 Aug	Cuneo, Piedmont, Italy	37	Right	1 female

Eye examinations showed thin, white nematode(s) on the conjunctival fornix of the affected eye. Nematodes were removed with a forceps after local anesthesia (1% novocaine) was administered. The nematodes were stored in 70% ethanol until they were morphologically identified and analyzed. After the parasites were removed from the eyes, antimicrobial eye drops were prescribed for ≈7 days. Ocular symptoms disappeared within 2–3 days.

**Figure 2:**
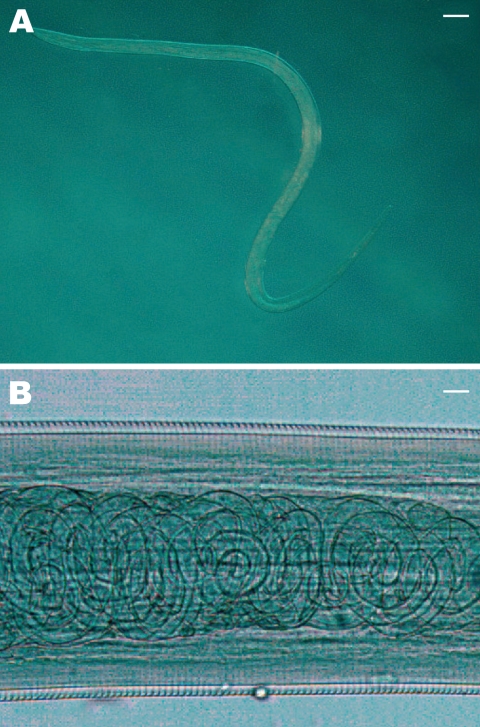
A) Female *Thelazia callipaeda* isolated from patient 4. The posterior end is on the left and the anterior end is on the right (magnification × 200). Scale bar = 500 μm. B) *T*. *callipaeda* mature first-stage larvae in the distal uterus (magnification ×100). Scale bar = 30 μm.

Collected nematodes were identified based on morphologic keys (*13*,*14*). *T*. *callipaeda* nematodes have a serrated cuticle (Figure 2, **panel A**), buccal capsule, mouth opening with a hexagonal profile, and 6 festoons. Adult females are characterized by the position of the vulva, located anterior to the esophagus-intestinal junction, whereas males have 5 pairs of postcloacal papillae. To confirm morphologic identification, specimens from patients 2 and 4 were analyzed as previously described (*11*). Genomic DNA was isolated from each nematode, and a partial sequence of the mitochondrial cytochrome c oxidase subunit 1 (*cox*1, 689 bp) gene was amplified by PCR. Amplicons were purified by using Ultrafree-DA columns (Amicon; Millipore, Bedford, MA, USA) and sequenced by using an ABI-PRISM 377 system and a Taq DyeDeoxyTerminator Cycle Sequencing Kit (Applied Biosystems, Foster City, CA, USA). Sequences were determined in both directions and aligned by using the ClustalX program (*15*). Alignments were verified visually and compared with sequences available for the *cox*1 gene of *T*. *callipaeda* (GenBank accession nos. AM042549–556).

A total of 6 adult nematodes were morphologically identified as *T*. *callipaeda* (Table). A mature female nematode (patient 4) had embryonated eggs in the proximal uterus and larvae in the distal uterus (Figure 2, **panel B**). This suggested that a male worm was present, which had been rubbed out of the eye before symptoms occurred or had remained undetected. Sequences obtained from nematodes were identical to the sequence of haplotype 1 of *T*. *callipaeda* (GenBank accession no. AM042549) (*11*).

## Conclusions

We report human infection by *T*. *callipaeda* in Italy and France in the same area where canine thelaziasis had been reported. These infections highlight the importance of including this arthropod-borne disease in the differential diagnoses of bacterial or allergic conjunctivitis. All cases of human thelaziasis were reported during the summer months (June–August), which is the period of *T*. *callipaeda* vector activity (late spring to fall in southern Europe) (*7*). The seasonality of human thelaziasis may impair correct etiologic diagnosis of this disease because spring and summer are the seasons in which allergic conjunctivitis (e.g., by pollens) occurs most frequently. This finding is particularly important when infections are caused by small larval stages that are difficult to detect and identify. Furthermore, clinical diagnosis of human thelaziasis is difficult if only small numbers of nematodes are present because clinical signs related to an inflammatory response mimic allergic conjunctivitis, especially when they are associated with developing third- or fourth-stage larvae.

Untimely or incorrect treatment of the infection may result in a delay in recovery, mainly in children and the elderly, who are most likely to be exposed to infection by the fly. Although treatment for canine infection with *T*. *callipaeda* with topical organophosphates, 1% moxidectin, or a formulation containing 10% imidacloprid and 2.5% moxidectin is effective, mechanical removal of parasites in humans remains the only curative option (*3*). Thus, prevention of human thelaziasis should include control of the fly vector by use of bed nets to protect children while they are sleeping and by keeping their faces and eyes clean. Genetic identification of haplotype 1 has shown that this is the only haplotype circulating in animals (i.e., dogs, cats, and foxes) in Europe (*11*). This finding confirms the metazoonotic potential of *Thelazia* spp. infection and the need to treat infected domestic animals, which may act as reservoirs for human infection.
